# Regulatory T cells require peripheral CCL2-CCR2 signaling to facilitate the resolution of medication overuse headache-related behavioral sensitization

**DOI:** 10.1186/s10194-024-01900-5

**Published:** 2024-11-11

**Authors:** Sun Ryu, Jintao Zhang, Roli Simoes, Xuemei Liu, Zhaohua Guo, Li Feng, Jacqueline Unsinger, Richard S. Hotchkiss, Yu-Qing Cao

**Affiliations:** 1 Department of Anesthesiology, Washington University in St. Louis School of Medicine, St. Louis, MO 63110 USA; 2 Washington University Pain Center, Washington University in St. Louis School of Medicine, St. Louis, MO 63110 USA; 3 Department of Medicine, Washington University in St. Louis School of Medicine, St. Louis, MO 63110 USA; 4 Department of Surgery, Washington University in St. Louis School of Medicine, St. Louis, MO 63110 USA

**Keywords:** Medication overuse headache, Facial mechanical hypersensitivity, C-C motif ligand 2 (CCL2), C-C motif chemokine receptor 2 (CCR2), Calcitonin gene-related peptide (CGRP), Regulatory T (Treg) cell, Low-dose interleukin-2 (LD-IL-2)

## Abstract

**Background:**

Medication overuse headache (MOH) is the most common secondary headache disorder, resulting from chronic and excessive use of medication to treat headaches, for example, sumatriptan. In a recent study, we have shown that the peripheral C-C motif ligand 2 (CCL2), C-C motif chemokine receptor 2 (CCR2) and calcitonin-gene-related peptide (CGRP) signaling pathways interact with each other and play critical roles in the development of chronic migraine-related behavioral and cellular sensitization. In the present study, we investigated whether CCL2-CCR2 and CGRP signaling pathways play a role in the development of sumatriptan overuse-induced sensitization, and whether they are involved in its resolution by the low-dose interleukin-2 (LD-IL-2) treatment.

**Methods:**

Mice received daily sumatriptan administration for 12 days. MOH-related behavioral sensitization was assessed by measuring changes of periorbital mechanical thresholds for 3 weeks. CCL2-CCR2 and CGRP signaling pathways were inhibited by targeted gene deletion or with an anti-CCL2 antibody. Ca^2+^-imaging was used to examine whether repetitive sumatriptan treatment enhances CGRP and pituitary adenylate cyclase–activating polypeptide (PACAP) signaling in trigeminal ganglion (TG) neurons. LD-IL-2 treatment was initiated after the establishment of sumatriptan-induced sensitization. Immunohistochemistry and flow cytometry analyses were used to examine whether CCL2-CCR2 signaling controls regulatory T (Treg) cell proliferation and/or trafficking.

**Results:**

CCL2, CCR2 and CGRPα global KO mice exhibited robust sumatriptan-induced behavioral sensitization comparable to wild-type controls. Antibody neutralization of peripheral CCL2 did not affect sumatriptan-induced behaviors either. Repeated sumatriptan administration did not enhance the strength of CGRP or PACAP signaling in TG neurons. Nevertheless, LD-IL-2 treatment, which facilitated the resolution of sumatriptan-induced sensitization in wild-type and CGRPα KO mice, was completely ineffective in mice with compromised CCL2-CCR2 signaling. In CCL2 KO mice, we observed normal LD-IL-2-induced Treg expansion in peripheral blood, but the increase of Treg cells in dura and TG tissues was significantly reduced in LD-IL-2-treated CCL2 KO mice relative to wild-type controls.

**Conclusions:**

These results indicate that the endogenous CCL2-CCR2 and CGRP signaling pathways are not involved in sumatriptan-induced behavioral sensitization, suggesting that distinct molecular mechanisms underlie chronic migraine and MOH. On the other hand, peripheral CCL2-CCR2 signaling is required for LD-IL-2 to reverse chronic headache-related sensitization.

**Graphical abstract:**

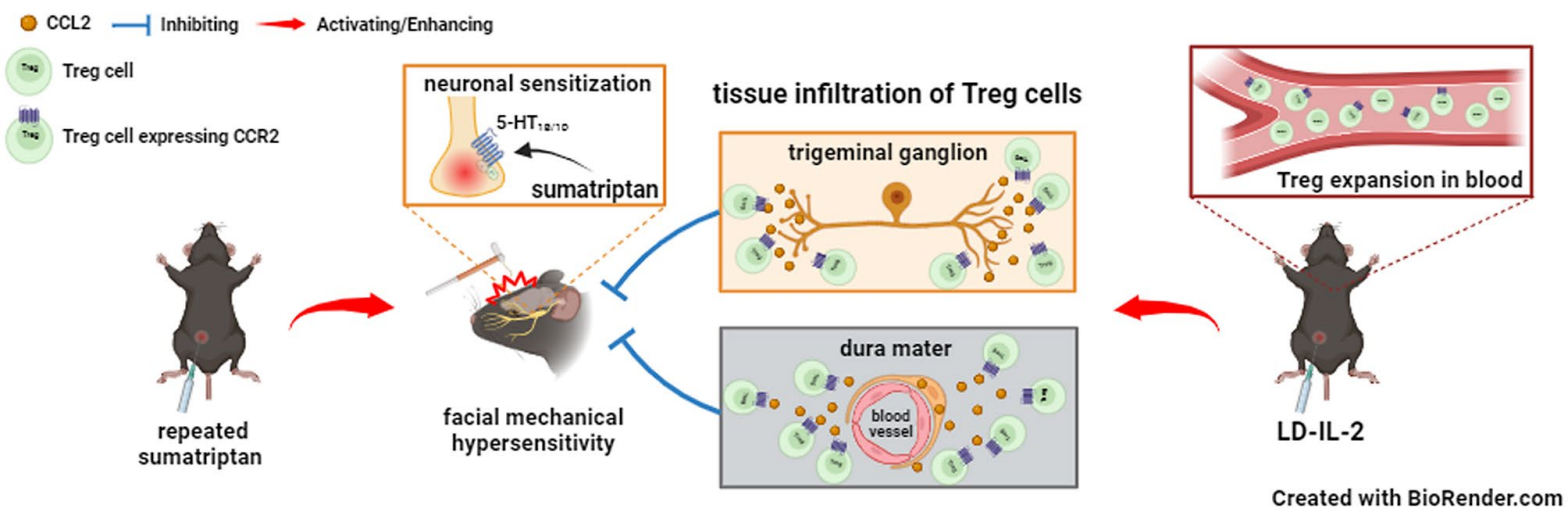

## Background

Medication overuse headache (MOH) is the most common secondary headache disorder, resulting from chronic and excessive use of medication to treat headaches [1, 2]. These patients experience more than 15 headache days per month, and many are refractory to preventive treatments. Withdrawal from medication overuse is often necessary to alleviate MOH. However, this may transiently exacerbate the headache and discourage drug discontinuation. Although MOH is associated with substantial disability and reductions in quality of life, *t*he pathophysiology of MOH is not well understood, hindering the development of MOH-specific treatments.

It is well documented that overuse of triptan family of migraine abortive drugs results in MOH [1, 2]. In rodent models, repeated sumatriptan administration causes behavioral sensitization resulting from changes in primary afferent neurons, central headache circuits as well as the descending pain modulation pathway [1]. Intradermal injection of sumatriptan activates serotonin 1B and 1D (5-HT_1B/1D_) receptors on primary afferent neurons, thereby inducing protein kinase Cε-dependent type I hyperalgesic priming [3, 4]. Chronic systemic administration of sumatriptan results in acute periorbital mechanical hypersensitivity indicative of central sensitization as well as hyperalgesic priming, a reduction of response thresholds to migraine triggers after the cessation of sumatriptan and the resolution of acute sensitization [5, 6]. Sumatriptan-induced hyperalgesic priming may be mechanistically related to withdrawal headaches during the acute phase of drug discontinuation and/or the transformation from episodic to chronic headache. In rats, the number of dural afferent neurons that express neuropeptide calcitonin gene-related peptide (CGRP) is increased after chronic sumatriptan treatment [5], and pharmacologically blocking of the CGRP signaling pathway prevents stress- or nitric oxide donor-induced behavioral sensitization after chronic sumatriptan exposure [7, 8]. Conversely, CGRP receptor antagonist olcegepant does not reverse MOH-related sensitization after chronic exposure of mice to both morphine and nitric oxide donor [9], suggesting that multiple signaling pathways are involved in the development of MOH. A more comprehensive understanding of the underlying mechanisms is needed to develop effective MOH therapy.

In a mouse model of chronic migraine, we have found that the peripheral C-C motif ligand 2 (CCL2), C-C motif chemokine receptor 2 (CCR2) and CGRP signaling pathways interact with each other and play critical roles in the development of chronic headache-related behavioral and cellular sensitization [10]. Many MOH patients have a long history of migraine headache that leads to overuse of abortive drugs, and medication overuse is one of the most important risk factors for the transformation from episodic to chronic migraine [11]. In this study, we asked whether repeated sumatriptan administration in mice alters the level of CCL2 and CCR2 mRNA expression in dura and trigeminal ganglion (TG), tissues that are strongly implicated in headache generation. Using both genetic and pharmacological approaches, we investigated whether CCL2-CCR2 and CGRP signaling pathways contribute to the development and maintenance of MOH-related behavioral and cellular sensitization.

Our previous work indicates that low-dose interleukin-2 (LD-IL-2) treatment reverses behavioral sensitization in mouse models of chronic migraine as well as sumatriptan-induced MOH through selective expansion/activation of regulatory T (Treg) cells at peripheral [12], suggesting that LD-IL-2 can be a potential therapy for patients with both chronic migraine and MOH. Treg cells belong to a subpopulation of CD3^+^CD4^+^ T cells that express transcription factor Foxp3 in the nucleus and interleukin-2 (IL-2) receptor α subunit CD25 on the plasma membrane [13, 14]. They possess far-ranging suppressive activities against various types of immune cells to maintain homeostasis [13, 14]. Given that all CD25^+^ cells in dura and TG express CCR2 [10], we used behavioral assay, immunohistochemistry and flow cytometry analysis to examine whether the CCL2-CCR2 signaling pathway is also functionally important for the therapeutic effect of LD-IL-2.

## Materials and methods

### Mice

All procedures were approved by the Institutional Animal Care and Use Committee at Washington University in St. Louis and were conducted in accordance with the PHS Policy on Humane Care and Use of Laboratory Animals. To avoid social isolation stress, all mice were group housed (2–5 per cage, same sex) in the animal facility of Washington University in St. Louis on a 12-hour light-dark cycle with constant temperature (23–24 °C), humidity (45–50%), and food and water *ad libitum*. All experiments were performed during the light phase (9 am – 4 pm). Adult mice (8–14 weeks old, both male and female) were used in the experiments. A total of 90 male and 170 female mice of various genotypes were used in this study. Estrous cycle analysis was not performed in female mice. In experiments using only wild-type mice, C57BL/6J or CD-1 mice were purchased from the Jackson Laboratory (Bar Harbor, ME) or Charles River (O’Fallon, MO), respectively.

#### CCL2 and CCR2 global knockout mice

CCL2 and CCR2 global knockout (KO) breeders on C57BL/6J background were purchased from the Jackson Laboratory (strains 004434 and 027619) and were crossed with wild-type C57BL/6J mice to generate heterozygous (HZ) breeders. Wild-type and KO mice were generated by crossing heterozygous breeders. CCR2 KO mice express enhanced green fluorescent protein (EGFP) from *Ccr2* alleles, abolishing endogenous CCR2 expression [15].

#### CGRPα knockout mice

Wild-type and CGRPα global KO mice on C57BL/6J background were generated by crossing HZ breeders purchased from the Mutant Mouse Resource & Research Center (MMRRC_036773_UNC, Chapel Hill, NC).

#### DEREG transgenic mice

DEREG (Depletion of regulatory T cell) breeders (strain 32050) on C57BL/6J background were purchased from the Jackson Laboratory. The DEREG mice contain a transgenic allele that expresses the diphtheria toxin receptor-EGFP (DTR-EGFP) fusion protein under the control of the genomic sequences that regulate the expression of endogenous FOXP3 [16]. DEREG breeders were crossed with C57BL/6J mice to generate HZ for experiments. We also crossed DEREG and CCL2 KO mice to generate DEREG_CCL2KO that express EGFP signal in Treg cells on CCL2 KO background.

Genotypes of genetically engineered mice were determined by polymerase chain reaction (PCR) of tail DNA as described previously [15–18].

### Mouse model of medication overuse headache

Mice were extensively handled by the experimenters for 2 weeks and were well-habituated to the test room and the test apparatus before baseline measurement. The experimenters were blinded to the genotype and the treatments mice received during data collection and analysis. To model MOH, mice received intraperitoneal (i.p.) injections of sumatriptan succinate (0.6 mg/kg, Fresenius Kabi, Lake Zurich, IL [12]), once every day for 12 days. The withdrawal thresholds to mechanical stimuli on facial skin were measured at various time points.

One to three days before testing, mice were lightly anesthetized with isofluorane. The hair on the mouse forehead (above and between the eyes) was shaved and trimmed with a Multi-Groom Ultra Precise Beard Styler trimmer Groomer (Philips, PHMG1100/16, Cambridge MA). On the test day, the experimenters gently held the mouse on the palm with minimal restraint and applied the calibrated von Frey filament perpendicularly to the shaved skin, causing the filament to bend for 5 s. A positive response was determined by the following criteria as in previous studies: mouse vigorously stroked its face with the forepaw, head withdrawal from the stimulus, or head shaking [12]. The up-down paradigm was used to determine the 50% withdrawal threshold [19].

In some experiments, after the facial mechanical threshold returned to baseline level, mice received 2 daily injections of nitroglycerin (NTG, 0.1 mg/kg, i.p.). Facial mechanical thresholds were measured 1 day later to assess repeated sumatriptan-induced hyperalgesic priming.

### Drug treatments

On the days that the mouse behaviors were tested, all drugs were injected after the completion of the behavioral assays. Sumatriptan succinate (Fresenius Kabi, Melrose Park, IL) was freshly diluted from a stock solution (12 mg/ml, stored at 4^o^C) with saline before each injection (0.6 mg/kg, i.p.). The NTG stock solution (10% in propylene glycol, SDM27, Copperhead Chemical, Tamaqua, PA) was stored in airtight glass vials at 4^o^C and was freshly diluted with saline before each injection. Mice received 2 daily i.p. injections of NTG (0.1 mg/kg, in saline with 0.01% propylene glycol) to assess hyperalgesic priming.

To neutralize the endogenous CCL2, mice received i.p. injections of the anti-mouse CCL2 antibody (CCL2 ab, 200 µg/mouse, BE0185; BioXcell, West Lebanon, NH [10]) every 4–7 days. The control group received isotype-matched control immunoglobulin (IgG, 200 µg/mouse, BE0091; BioXcell) in parallel.

Recombinant mouse IL-2 (carrier-free, Biolegend, San Diego, CA) was freshly diluted from the stock (1 mg/ml aliquots at -80^o^C) every day. Mice received daily i.p. injections of saline or IL-2 (1 µg in 100 µl saline) for 6–12 days.

To deplete endogenous Treg cells, DEREG mice received i.p. injections of 0.5 µg diphtheria toxin (Sigma) in 100 µl saline for two consecutive days every 6 days [20, 21].

### RNA extraction, reverse transcription and quantitative polymerase chain reaction

Female CD-1 mice received i.p. saline or sumatriptan (0.6 mg/kg) injections once every days for 4 or 12 times. Mice were euthanized 1 day after the last sumatriptan administration. Dura and TG tissues were quickly removed, stored in RNAprotect Tissue reagent (Qiagen, Valencia, CA) and then homogenized using a Polytron homogenizer (Qiagen). Total RNA was isolated using a RNeasy mini Kit (Qiagen) and treated with DNase. Reverse transcription was performed using a High-Capacity cDNA Reverse Transcription Kit (Applied Biosystems, Foster City, CA) based on the manufacturer’s protocols.

The quantitative PCR (qPCR) reactions were performed with samples in triplicate on an ABI 7500 fast real-time PCR system using the Taqman Gene Expression Master Mix (Applied Biosystems) [10]. The primer Taqman probe for mouse CCL2 and CCR2 cDNAs (Mm00441242_m1 and Mm99999051_gH, Applied Biosystems) were used. The real-time qPCR reactions underwent 50 cycles; cycling conditions for these genes were as follows: 2 min (min) at 95 °C for denaturing, 3 s at 95 °C for annealing, and 30 s at 60 °C for extension. Duplicate samples without cDNA (no-template control) for each gene showed no contaminating DNA. Relative CCL2 and CCR2 mRNA levels were normalized to glyceraldehyde 3-phosphate dehydrogenase (GAPDH, Mm 99999915_g1, Applied Biosystems) and quantified by use of the comparative CT (ΔΔCT) method for calculating relative quantitation of gene expression.

### Primary culture of mouse trigeminal ganglion neurons and ratiometric Ca^2+^ imaging

Ratiometric Ca^2+^ imaging of TG neurons was performed as described in a previous study [22]. Briefly, after 12 daily injections of saline or sumatriptan, TG tissues were collected and were treated with 2.5 mg/ml collagenase IV for 10 min followed by 2.5 mg/ml trypsin at 37^o^C for 15 min. Cells were dissociated by triturating with fire-polished glass pipettes, resuspended in MEM-based culture medium containing 5% fetal bovine serum, 25 ng/ml nerve growth factor (R&D, Minneapolis, MN) and 10 ng/ml glial cell-derived neurotrophic factor (R&D), and seeded on Matrigel-coated coverslips. Ca^2+^ imaging was performed 2 days later. Each experiment contained neurons from at least 2 batches of culture.

Coverslips containing cultured TG neurons were incubated with HBSS/HEPES solution containing 2.5 µM fura-2 AM and 10% Pluronic F-68 (both from Molecular Probes, Eugene, OR) at 37^o^C for 45 min to load the ratiometric Ca^2+^ indicator. De-esterification of the dye was carried out by washing the coverslips 3 times with HBSS/HEPES solution and incubating the coverslips in HBSS/HEPES solution in the dark for an additional 15 min at 37^o^C. Neurons were used for Ca^2+^ imaging experiments within 1 h after Fura-2 loading.

Coverslips with fura-2 loaded neurons were placed in a flow chamber mounted on a Nikon TE2000S inverted epifluorescent microscope and were perfused with room temperature (RT) Tyrode’s solution (1 ml/min) containing (in mM): 130 NaCl, 2 KCl, 2 CaCl_2_, 2 MgCl_2_, 25 Hepes, 30 glucose, pH 7.3–7.4 with NaOH, and 310 mosmol/kgH_2_O. Healthy neurons were chosen based on the differential interference contrast images. Fura-2 was alternately excited by 340 and 380 nm light (Sutter Lambda LS, Sutter Instrument, Navato, CA) and the emission was detected at 510 ± 20 nm by a UV-transmitting 20x objective (N.A. 0.75) and a prime BSI back illuminated sCMOS camera (Photometrics, Tuscon, AZ). The frame capture period was 50 milliseconds at 1.5 s interval. Metamorph software (Molecular Devices, San Jose, CA) was used for controlling and synchronizing the devices as well as image acquisition and analysis. After a 2–3 min baseline measurement in Tyrode’s solution, neurons were perfused with 50 nM PACAP_1 − 38_ (pituitary adenylate cyclase–activating polypeptide 1–38, Tocris, Ellisville, MO) for 1 min followed by washing with Tyrode’s for 4 min. Subsequently, the coverslip was incubated with 3 µM human CGRPα (Tocris) for 1 min followed by washing with Tyrode’s for 4 min. CGRP and PACAP were freshly diluted from the stock (aliquots at -80^o^C) in Tyrode’s solution every day.

Regions of interest (ROIs) encompassing individual neurons were defined a priori. The ratio of fluorescence excited by 340 nm divided by fluorescence excited by 380 nm (R_340/380_) was determined on a pixel-by-pixel basis and was averaged for each ROI. An additional background area was recorded in each field for off-line subtraction of background fluorescence. Peak responses were determined by calculating the relative increase in R_340/380_ above baseline (F_0_, the average R_340/380_ during the 2–3 min baseline measurement). A ΔF/F_0_ > 20% was set as the threshold for a response.

### Blood collection, antibody staining and flow cytometry

Mice used for flow cytometry analysis were not used in behavioral tests. Female DEREG and DEREG_CCL2KO mice received 5 daily injections of LD-IL-2. About 100 µl blood was collected from each mouse by submandibular bleeding. Cell suspensions were stained with the antibodies (all from Biolegend) that recognize mouse CD3ε (clone 145-2C11), CD4 (clone GK1.5 and RM4-5), and CD25 (clone PC61) for 20 min. The frequency of individual cell subpopulations was determined via flow cytometric analysis. Data were collected with FACScan (Becton Dickinson, Franklin Lakes, NJ) and analyzed with the CellQuest Pro (Becton Dickinson) and Rainbow X Alias (Cytek, Freemont, CA) software.

### Tissue preparation, immunohistochemistry, and image analysis

Immunohistochemistry approach was used to quantify Treg cells in the dura because the distribution of Treg cells is highly uneven in different regions of the dura. Immunohistochemistry analysis allows us to focus on Treg cells in dura areas adjacent to the middle meningeal artery (MMA). This increases the sensitivity of detecting the effect of CCL2-CCR2 signaling on Treg cell infiltration from peripheral blood to tissues. Moreover, dural afferent neurons innervating the MMA region have been implicated in headache pathophysiology by many studies, Treg cells in this region likely contribute to the neuroimmune interactions that regulate dural afferent activities and headache susceptibility. We also used immunohistochemistry to quantify Treg cells in TG. This allowed us to easily distinguish Treg cells localized within the TG tissues from those that are localized in the membrane surrounding the TG tissues.

Immunohistochemistry and image analysis were performed as previously described [10, 12], with experimenters blinded to the treatments mice received. Briefly, mice were euthanized with i.p. injection of barbiturate (200 mg/kg) and were transcardially perfused with warm 0.1 M phosphate buffered saline (PBS, pH 7.2) followed by cold 4% formaldehyde in 0.1 M phosphate buffer (PB, pH 7.2) for fixation. TG tissues were collected and sectioned at 15 μm in the transverse plane on a cryostat, collected on Superfrost Plus glass slides in sequence and stored at -20 °C.

One in every 3 TG sections were processed for each immunohistochemistry experiment. The sections were dried at RT, washed three times in 0.01 M PBS, and incubated in blocking buffer consisting 0.01 M PBS, 10% normal goat serum (NGS), and 0.3% triton X-100 for 1 h at RT. Sections from DEREG and DEREG_CCL2KO mice were then incubated with the chicken anti-EGFP antibody (1:1000 dilution in blocking buffer, AVES Lab, Davis, CA) in a humidity chamber at 4 °C overnight. After 6 washes (5 min each) in washing buffer containing 0.01 M PBS with 1% NGS and 0.3% triton, sections were incubated with blocking buffer for 1 h, followed by the incubation with AlexaFluor 488-conjugated secondary antibody (1:1000 dilution in blocking buffer, Invitrogen, Waltham, MA) at RT for 1 h. After washing off the antibody, sections were rinsed with PBS, cover-slipped using Fluoromount-G Slide Mounting Medium (Electron Microscopy, Hatfield, PA), sealed with nail polish, and stored at 4 °C. Sections from CCR2 HZ mice were incubated overnight with AlexaFluor 594-conjugated anti-mouse CD25 antibody (1:50, clone PC61, Biolegend) in a humidity chamber at 4 °C.

Immunofluorescence was observed through a 40x objective on a Nikon TE2000S inverted epifluorescence microscope. To quantify Treg cells in TG, all CD25^+^ cells in individual sections from CCR2 HZ mice and all EGFP^+^ cells in individual sections from DEREG_WT and DEREG_CCL2KO mice were counted, and the number was multiplied by 3 to obtain the total number of cells per TG in each mouse. Cells that were localized in the membranes surrounding the TG tissues were not included in the quantification.

Dura was carefully dissected from the skull using forceps after 4 h fixation, washed 3 times in 0.01 M PBS and stained as whole-mount in a 48-well plate. The blocking and washing buffers used for dura staining contained 0.1% triton. Treg cells in the area adjacent to the MMA were quantified.

### Statistical analysis

For behavioral experiments, power analysis was conducted to estimate sample size with > 80% power to reach a significance level of 0.05. For immunohistochemistry and flow cytometry experiments, sample sizes were estimated based on our prior experience.

All data were reported as mean ± standard error of the mean. The Shapiro–Wilk test was used to check data normality. Statistical significance within or between experimental groups was assessed by two-tailed t test, analysis of variance (ANOVA, one-way or two-way, with or without repeated-measures [RM]) with post hoc Bonferroni test or Student-Newman-Keuls test, where appropriate, using Origin (OriginLab Corporation, Northampton, MA) or Statistica (StatSoft Inc, Tulsa, OK). Differences with *p* < 0.05 were considered statistically significant. The statistical analysis for individual experiments was described in figure legends or in Table 1.

**Table 1:** Statistical analysis in individual experiments

Figure	Statistical analysis	Post hoc analysis
Figure 1B	^*^p < 0.05, two-tailed t-test	
Figure 2B	One-way RM ANOVA: Wild-type: *p* < 0.001, F_[8,72]_ = 354.13 CCL2 KO: *p* < 0.001, F_[8,64]_ = 31.14	Student–Newman–Keuls test: ^*###*^*p* < 0.001, compared with the day 1 baseline threshold within individual groups, respectively
Figure 2C	One-way RM ANOVA: Wild-type: *p* < 0.01, F_[8,48]_ = 23.73 CCR2 KO: *p* < 0.01, F_[8,48]_ = 27.71	Student–Newman–Keuls test: ^*###*^*p* < 0.001, compared with the day 1 baseline threshold within individual groups, respectively
Figure 2D	One-way RM ANOVA: Day 1–20: wild-type: *p* < 0.01, F_[8,64]_ = 81.57 CCL2 KO: *p* < 0.01, F_[8,48]_ = 23.87 Day 20–25: wild-type: *p* < 0.01, F_[2,16]_ = 45.34 CCL2 KO: *p* < 0.01, F_[2,12]_ = 5.15	Student–Newman–Keuls test: ^###^*p* < 0.001, compared with the day 1 baseline threshold within individual groups. ^!!!^*p* < 0.001, compared with the day 20 threshold within individual groups.
Figure 2E	One-way RM ANOVA: Day 1–20: wild-type: *p* < 0.01, F_[8,42]_ = 43.89 CCR2 KO: *p* < 0.01, F_[8,42]_ = 80.54 Day 20–25: wild-type: *p* < 0.001, F_[2,14]_ = 81.73 CCR2 KO: *p* < 0.001, F_[2,12]_ = 6.38	Student–Newman–Keuls test: ^###^*p* < 0.001, compared with the day 1 baseline threshold within individual groups. ^!!!^*p* < 0.001, compared with the day 20 threshold within individual groups.
Figure 2F	One-way RM ANOVA: Day 1–20: Control IgG: *p* < 0.01, F_[8,56]_ = 24.14 CCL2 ab: *p* < 0.01, F_[8,56]_ = 32.07 Day 20–25: Control IgG: *p* < 0.01, F_[2,14]_ = 70.02 CCL2 ab: *p* < 0.01, F_[2,14]_ = 53.82	Student-Newman–Keuls test: ^*###*^*p* < 0.001, compared with the day 1 baseline threshold within individual groups, respectively. ^!!!^*p* < 0.001, compared with the day 20 threshold within individual groups, respectively.
Figure 3B	One-way RM ANOVA: Day 1–20: wild-type: *p* < 0.01, F_[8,64]_ = 81.57 CGRPα KO: *p* < 0.01, F_[8,64]_ = 38.84 Day 20–25: wild-type: *p* < 0.01, F_[2,16]_ = 45.34 CGRPα KO: *p* < 0.01, F_[2,16]_ = 45.34	Student–Newman–Keuls test: ^###^*p* < 0.001, compared with the day 1 baseline threshold within individual groups. ^!!!^*p* < 0.001, compared with the day 20 threshold within individual groups.
Figure 3C	One-way RM ANOVA: Day 1–20: wild-type: *p* < 0.01, F_[8,42]_ = 43.89 CGRPα KO: *p* < 0.01, F_[8,42]_ = 122.9 Day 20–25: wild-type: *p* < 0.001, F_[2,14]_ = 81.73 CGRPα KO: *p* < 0.001, F_[2,12]_ = 93.36	Student–Newman–Keuls test: ^###^*p* < 0.001, compared with the day 1 baseline threshold within individual groups. ^!!!^*p* < 0.001, compared with the day 20 threshold within individual groups.
Figure 5B	Two-way RM ANOVA: *p* < 0.001 Group: F_[1, 128]_ = 27.16 Time: F_[8, 128]_ = 20.95 Group x time interaction: F_[8, 128]_ = 9.03 One-way RM ANOVA: Wild-type: *p* < 0.001, F_[8,64]_ = 8.27 CCL2 KO: *p* < 0.001, F_[8,64]_ = 22.60	Student–Newman–Keuls test: ^*^p < 0.05, ^***^p < 0.001, wild-type versus CCL2 KO ^###^*p* < 0.001, compared with the day 1 baseline threshold in wild-type group; ^††^*p* < 0.01, ^†††^*p* < 0.001, compared with the baseline threshold in CCL2 KO group.
Figure 5C	Two-way RM ANOVA: *p* < 0.001 Group: F_[1,104]_ = 24.22 Time: F_[8,104]_ = 12.14 Group x time interaction: F_[8,104]_ = 3.81 One-way RM ANOVA: Wild-type: *p* < 0.001, F_[8,48]_ = 3.29 CCR2 KO: *p* < 0.001, F_[8,56]_ = 16.02	Student–Newman–Keuls test: ^*^p < 0.05, ^**^p < 0.01, wild-type versus CCR2 KO ^###^*p* < 0.001, compared with the day 1 baseline threshold in wild-type group; ^†††^*p* < 0.001, compared with the baseline threshold within the CCR2 KO group.
Figure 5D	Two-way RM ANOVA: *p* < 0.001 Group: F_[1,13]_ = 37.59 Time: F_[9,117]_ = 58.54 Group x time interaction: F_[9,117]_ = 19.74 One-way RM ANOVA: Control IgG: *p* < 0.001, F_[9,54]_ = 42.27 CCL2 ab: *p* < 0.001, F_[9,63]_ = 37.34	Student–Newman–Keuls test: ^***^p < 0.001, control IgG versus CCL2 ab groups ^††^*p* < 0.01, ^†††^*p* < 0.001, compared with the day 1 baseline threshold in control IgG group ^###^*p* < 0.001, compared with the day1 baseline threshold in CCL2 ab group ^!!!^*p* < 0.001, compared with the day 20 threshold in the CCL2 ab group
Figure 5E	Two-way RM ANOVA: *p* < 0.001 Group: F_[1,12]_ = 126.0 Time: F_[9,108]_ = 63.84 Group x time interaction: F_[9,108]_ = 24.88 One-way RM ANOVA: Control IgG: *p* < 0.001, F_[9,54]_ = 30.41 CCL2 ab: *p* < 0.001, F_[9,54]_ = 102.7	Student–Newman–Keuls test: ^***^p < 0.001, control IgG versus CCL2 ab groups ^†††^*p* < 0.001, compared with the day 1 baseline threshold in control IgG group ^###^*p* < 0.001, compared with the day 1 baseline threshold in the CCL2 ab group ^!!!^*p* < 0.001, compared with the day 20 threshold in the CCL2 ab group
Figure 6A	one-way RM ANOVA: Wild-type: *p* < 0.01, F_[8,72]_ = 44.35 CGRPα KO: *p* < 0.01, F_[8,48]_ = 122.9	Student–Newman–Keuls test: ^###^*p* < 0.001, ^†††^*p* < 0.001, compared with the baseline threshold within the wild-type and CGRPα KO groups, respectively.
Figure 6B	one-way RM ANOVA: Wild-type: *p* < 0.001, F_[8,48]_ = 6.96 CGRPα KO: *p* < 0.001, F_[8,64]_ = 11.41	Student–Newman–Keuls test: ^###^*p* < 0.001, ^†††^*p* < 0.001, compared with the baseline threshold within the wild-type and CGRPα KO groups, respectively.
Figure 7A	Two-way RM ANOVA: *p* < 0.001 Group: F_[1,14]_ = 77.27 Time: F_[7,98]_ = 74.37 Group x time interaction: F_[7,98]_ = 19.93 One-way RM ANOVA: saline: *p* < 0.001, F_[7,49]_ = 23.20 DT: *p* < 0.001, F_[7,49]_ = 179.5	Student–Newman–Keuls test: ^***^p < 0.001, saline versus DT groups. ^#^*p* < 0.05, ^###^*p* < 0.001, compared with the day 1 threshold in the saline group ^†††^*p* < 0.001, compared with the day 1 threshold in the DT group.
Figure 7D-E	^**^p < 0.01, two-tailed t-test	
Figure 8A	Two-way ANOVA: genotype: *p* < 0.01 treatment: *p* < 0.001 genotype x treatment: *p* < 0.001	post hoc t test with Bonferroni correction: ^*^p < 0.05, ^**^p < 0.01
Figure 8B	Two-way ANOVA: genotype: *p* < 0.01 treatment: *p* < 0.001 genotype x treatment: *p* < 0.05	post hoc t test with Bonferroni correction: ^*^p < 0.05, ^**^p < 0.01
Figure 8D	Two-way RM ANOVA: genotype: *p* = 0.049, F_[1,10]_ = 5.04 treatment: *p* < 0.001, F_[1,10]_ = 74.21 genotype x treatment: *p* = 0.19, F_[1,10]_ = 1.951	post hoc t test with Bonferroni correction: ^**^p < 0.01, ^***^p < 0.001
Figure 8E	Two-way RM ANOVA: genotype: *p* = 0.028, F_[1,10]_ = 6.55 treatment: *p* < 0.001, F_[1,10]_ = 51.17 genotype x treatment: *p* = 0.093, F_[1,10]_ = 3.46	post hoc t test with Bonferroni correction: ^*^p < 0.05, ^***^p < 0.001

## Results

### The endogenous CCL2-CCR2 signaling pathway is not required for repeated sumatriptan-induced behavioral sensitization

**Fig. 1:**
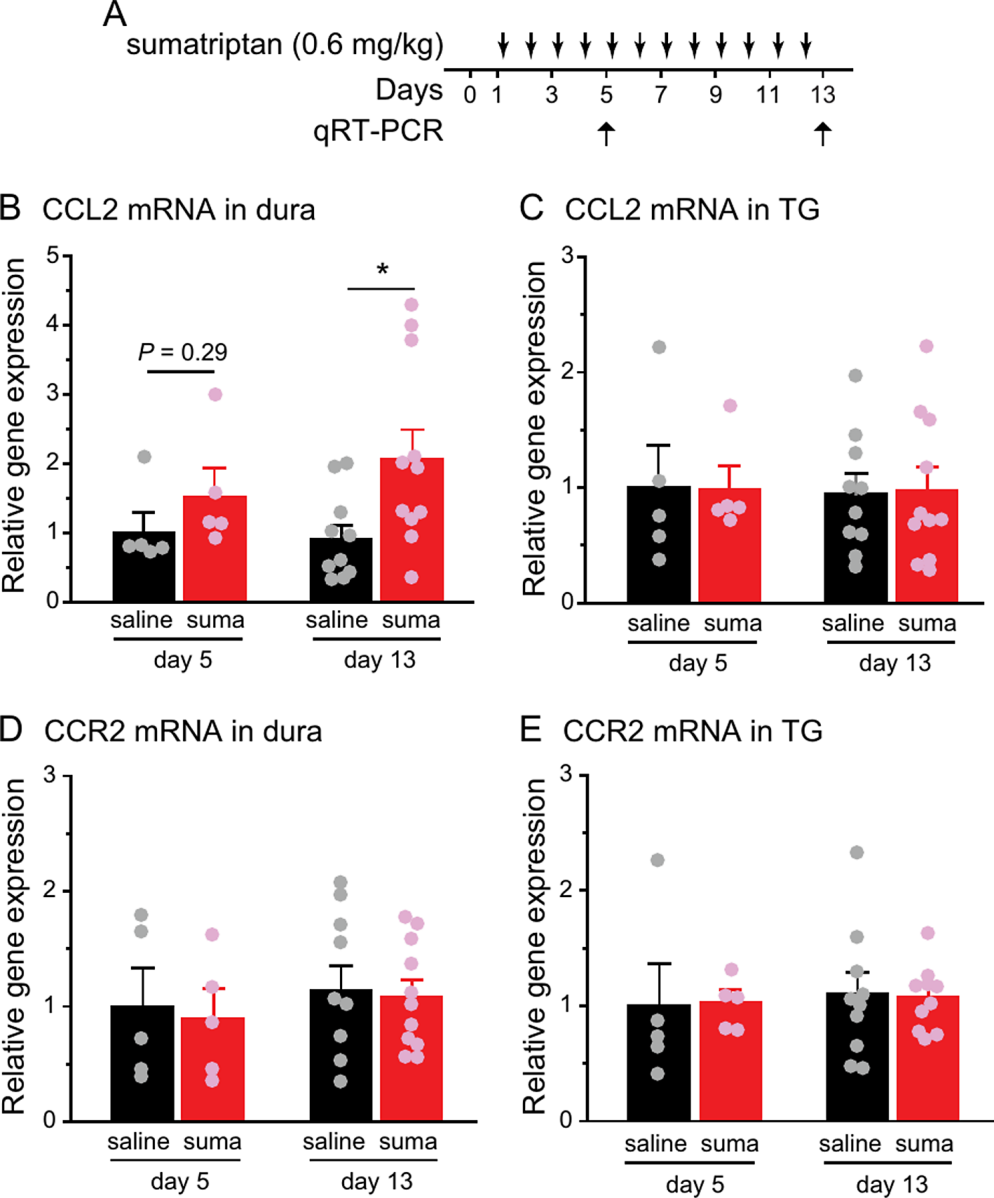
CCL2 and CCR2 mRNA expression in dura or TG after repeated sumatriptan administration. **A** Timeline of experiments. **B-C** Relative CCL2 mRNA expression levels in dura **(B)** and TG **(C)** of female CD-1 mice after 4 or 12 daily i.p. injections of saline or sumatriptan (suma, *n* = 5–11/group). The abundance of CCL2 mRNA was normalized to that of GAPDH in individual samples. ^*^p < 0.05, two-tailed t-test. **D-E** Relative CCR2 mRNA expression levels in dura **(D)** and TG **(E)** after 4 or 12 daily injections of saline or sumatriptan (same mice as in **B-C**). The abundance of CCR2 mRNA was normalized to that of GAPDH in individual samples

MOH results from the chronic and excessive use of anti-migraine drugs such as sumatriptan [2]. In rodents, prolonged exposure to sumatriptan leads to persistent facial skin hypersensitivity, indicating the development of MOH-related central sensitization [5, 6, 12]. Given the critical role of peripheral CCL2-CCR2 signaling pathway in nitric oxide- and repetitive stress-induced headache chronification [10], we asked whether repeated sumatriptan administration alters the level of CCL2 and CCR2 mRNA in peripheral tissues that are strongly implicated in headache generation and chronification (Fig. 1A). In female CD-1 mice, 12 daily sumatriptan injections increased the level of CCL2 mRNA in the dura but not in TG (Fig. 1B-C). The expression level of CCR2 mRNA in dura or TG was not altered by repeated sumatriptan administration (Fig. 1C-E).

**Fig. 2:**
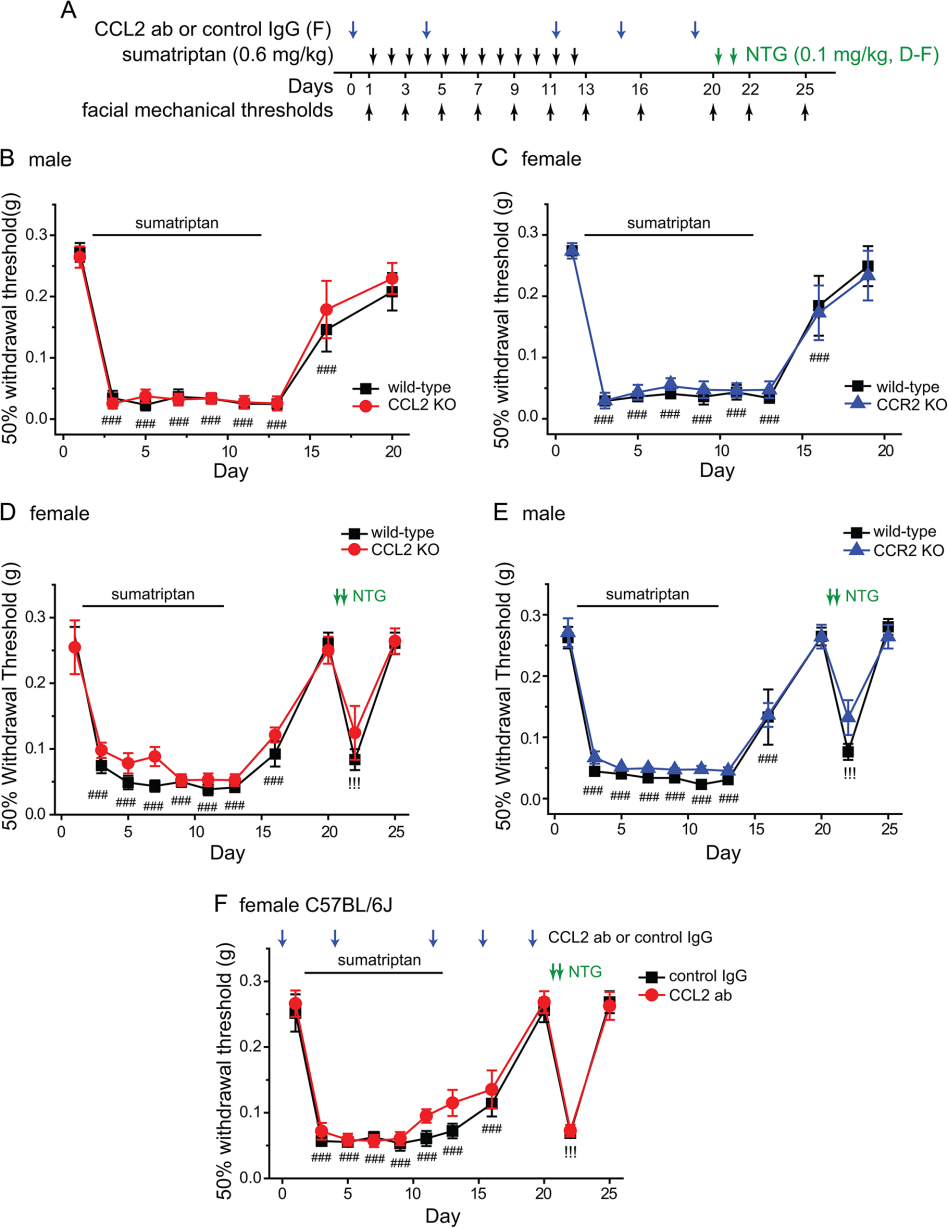
CCL2 and CCR2 global KO mice still exhibit repeated sumatriptan-induced behavioral sensitization. **A** Timeline of experiments. Note that drugs were always injected after the completion of behavioral tests on the same day. **B-C** Sumatriptan-induced facial skin hypersensitivity was comparable in wild-type, male CCL2 KO, and female CCR2 KO mice (*n* = 6–9/group). ^###^*p* < 0.001, compared with the day 1 baseline threshold within individual groups. **D-E** Sumatriptan-induced acute sensitization and hyperalgesic priming were also comparable between wild-type, female CCL2, and male CCR2 global KO mice (*n* = 6–8/group). ^###^*p* < 0.001, compared with the day 1 baseline threshold within individual groups. ^!!!^*p* < 0.001, compared with the day 20 threshold within individual groups. **F** Repeated sumatriptan-induced acute sensitization and hyperalgesic priming were not altered by the CCL2 neutralizing antibody (*n* = 7/group). ^###^*p* < 0.001, compared with the day 1 baseline threshold within individual groups. ^!!!^*p* < 0.001, compared with the day 20 threshold within individual groups

To investigate whether endogenous CCL2-CCR2 signaling functionally contributes to the pathogenesis of MOH, we compared the responses of wild-type (C57BL/6J), CCL2 global KO, and CCR2 global KO mice to repeated sumatriptan administration (Fig. 2A). In both male and female wild-type mice, 2 daily injections of sumatriptan (0.6 mg/kg, i.p.) resulted in robust facial mechanical hypersensitivity (Fig. 2B-C, wild-type, day 3). This was maintained by subsequent sumatriptan administration and was resolved one week after the cessation of sumatriptan (Fig. 2B-C, wild-type groups). Surprisingly, the magnitude and duration of sumatriptan-induced facial skin hypersensitivity in male CCL2 KO and female CCR2 KO mice were indistinguishable from those of wild-type mice (Fig. 2B-C, CCL2 KO and CCR2 KO groups). Repeated sumatriptan administration also induced similar behavioral responses in wild-type, female CCL2 KO and male CCR2 KO mice (Fig. 2D-E). After the facial mechanical thresholds returned to baseline level, 2 daily injections of NTG (0.1 mg/kg, i.p.), a nitric oxide donor and a reliable migraine trigger in humans, re-established facial skin hypersensitivity in wild-type mice (Fig. 2D-E, wild-type groups, day 22). Naïve C57BL/6J mice did not respond to the same NTG administration (data not shown [12]). Thus, repeated sumatriptan administration induces hyperalgesic priming, a reduction of the response thresholds to NTG and maybe other migraine triggers [5]. NTG injections also re-established facial skin hypersensitivity in female CCL2 KO and male CCR2 KO mice (Fig. 2D-E, day 22), indicating that genetic loss of CCL2 or CCR2 does not affect repeated sumatriptan-induced persistent sensitization and hyperalgesic priming.

Global gene deletion may induce compensatory changes that confound the phenotypic analysis. To address this possibility, we inhibited peripheral CCL2-CCR2 signaling in female C57BL/6J mice with a neutralizing CCL2 ab (200 µg/mouse, i.p.) before and during repeated sumatriptan administration. This did not affect the development and maintenance of acute behavioral sensitization (Fig. 2F, day 1–7). The resolution of sumatriptan-induced sensitization was not altered by the CCL2 ab either (Fig. 2F, day 12–20). After the facial mechanical thresholds returned to baseline level, 2 daily injections of NTG (0.1 mg/kg, i.p.) re-established the facial skin hypersensitivity in control IgG- as well as CCL2 ab-treated mice (Fig. 2F, day 22). Together, these results indicate that the endogenous CCL2-CCR2 signaling is not required for the development, maintenance, and resolution of sumatriptan-induced persistent sensitization and hyperalgesic priming.

### The CGRP signaling pathway does not contribute to repeated sumatriptan-induced acute sensitization and hyperalgesic priming

**Fig. 3:**
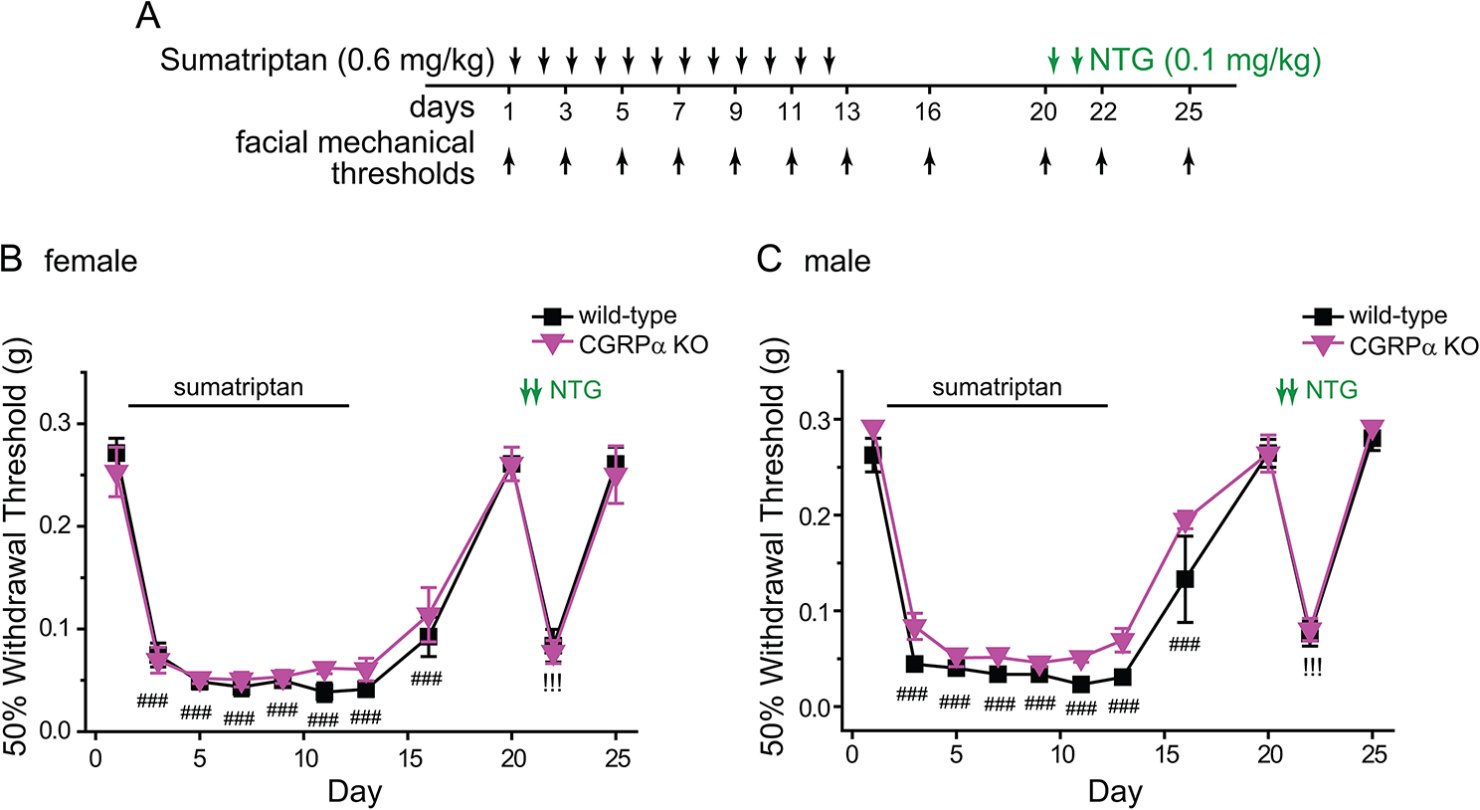
Repeated sumatriptan induces acute sensitization and hyperalgesic priming in CGRPα global KO mice. **A** Timeline of experiments. Note that sumatriptan and NTG were always injected after the completion of behavioral tests on the same day. **B-C** Both female (**B**) and male (**C**) CGRPα KO mice exhibited sumatriptan-induced facial skin hypersensitivity and hyperalgesic priming to that of wild-type control mice (*n* = 6–8/group). Wild-type mice in **B** and **C** are the same as mice in **2D** and **2E**, respectively. ^###^*p* < 0.001, compared with the day 1 baseline threshold within individual groups. ^!!!^*p* < 0.001, compared with the day 20 threshold within individual groups

Our previous work shows that the interaction between peripheral CCL2 and CGRP signal pathways plays a pivotal role in nitric oxide signaling-induced headache chronification [10]. In this study, we compared the behaviors of wild-type (C57BL/6J) and CGRPα global KO mice in response to repeated administration of sumatriptan (Fig. 3A). Both male and female CGRPα KO mice exhibited sumatriptan-induced acute sensitization and hyperalgesic priming indistinguishable from those of wild-type mice (Fig. 3B-C). We conclude that neither CCL2-CCR2 nor CGRP signaling pathway contributes to MOH-related behavioral sensitization resulting from repeated administration of sumatriptan.

**Fig. 4:**
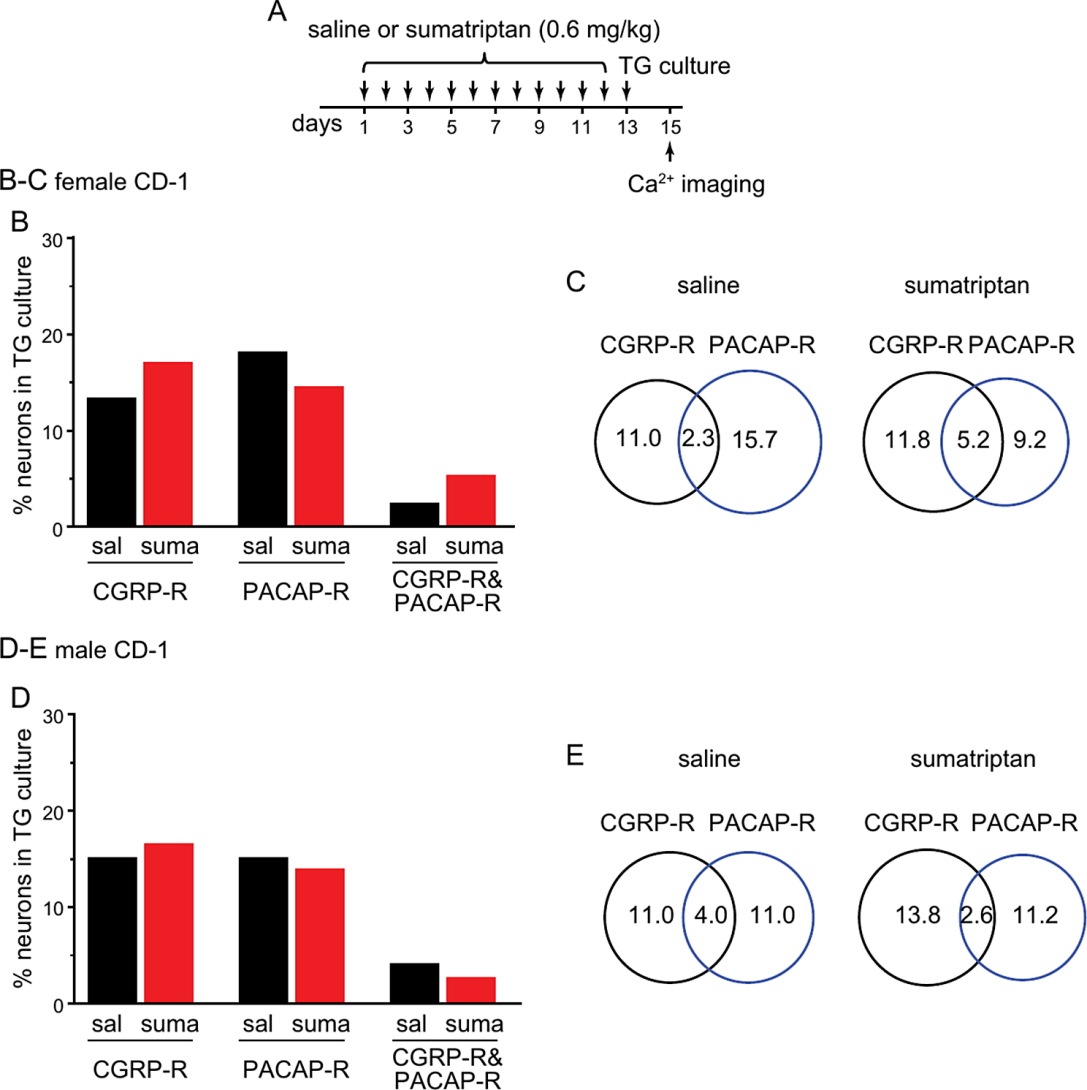
Repeated sumatriptan does not increase the number of CGRP-R and/or PACAP-R neurons in TG. **A** Timeline of experiments. **B** The percentages of CGRP-R, PACAP-R and CGRP-R&PACAP-R neurons in TG cultures from female CD-1 mice after repeated saline (sal) or sumatriptan (suma) administration (*n* = 4 mice/group, a total of 128 and 153 neurons were measured in saline and sumatriptan groups, respectively). **C** Venn diagrams of the percentages of CGRP-R and PACAP-R TG neurons in saline and sumatriptan groups (same neurons as in **B**), respectively. **D** The percentages of CGRP-R, PACAP-R and CGRP-R&PACAP-R neurons in TG cultures from female CD-1 mice after repeated saline or sumatriptan administration (*n* = 5 mice/group, a total of 100 and 195 neurons were measured in sal and suma groups, respectively). **E** Venn diagrams of the percentages of CGRP-R and PACAP-R TG neurons in saline and sumatriptan groups (same neurons as in **D**), respectively

We also investigated whether repeated sumatriptan administration enhances peripheral CGRP and/or PACAP signaling as in mouse models chronic migraine and post-traumatic headache [22]. To this end, we cultured TG neurons from female and male CD-1 mice after repeated saline or sumatriptan administration (Fig. 4A) and used ratiometric Ca^2+^ imaging to identify neurons that respond to neuropeptides CGRP and/or PACAP. Consistent with findings from behavioral assessments, the percentages of CGRP-responsive (CGRP-R) and PACAP-responsive (PACAP-R) neurons in TG cultures were comparable between saline and sumatriptan groups (Fig. 4B-E). The number of TG neurons that responded to both CGRP and PACAP (CGRP-R&PACAP-R) was not altered by repeated sumatriptan either (Fig. 4B-E). Thus, repeated sumatriptan administration does not increase the number of TG neurons that express functional CGRP and/or PACAP receptors in either male or female mice.

### LD-IL-2 treatment requires endogenous CCL2-CCR2 signaling to reverse headache-related behavioral sensitization

**Fig. 5:**
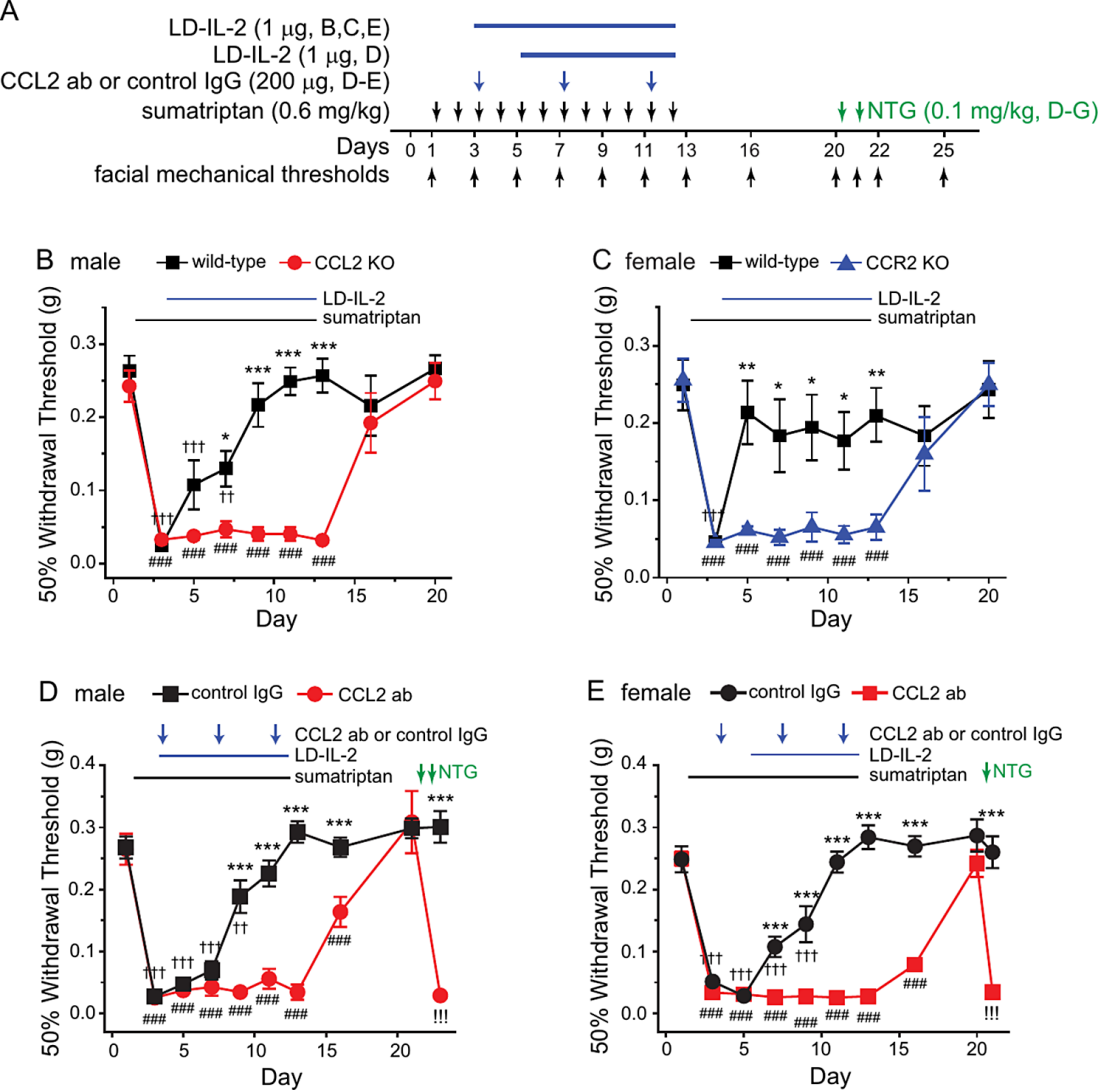
Loss of CCL2 or CCR2 abolishes the effect of LD-IL-2 on sumatriptan-induced behavioral sensitization. **A** Timeline of experiments. Note that all drugs were always injected after the completion of behavioral tests on the same day. **B** LD-IL-2 reversed sumatriptan-induced facial skin hypersensitivity in wild-type males but not CCL2 KO mice (*n* = 8/group). ^*^p < 0.05, ^***^p < 0.001, wild-type versus CCL2 KO; ^###^*p* < 0.001, compared with the day 1 baseline threshold within the wild-type group; ^††^*p* < 0.01, ^†††^*p* < 0.001, compared with the baseline threshold within the CCL2 KO group. **C** The effect of LD-IL-2 on sumatriptan-induced behavioral sensitization was absent in female CCR2 KO mice (*n* = 6–7/group). ^*^p < 0.05, ^**^p < 0.01, wild-type versus CCR2 KO; ^###^*p* < 0.001, compared with the day 1 baseline threshold within the wild-type group; ^†††^*p* < 0.001, compared with the baseline threshold within the CCR2 KO group. **D** CCL2 neutralizing antibody blocked the effect of LD-IL-2 on sumatriptan-induced behavioral sensitization in male C57BL/6J mice (*n* = 7–8/group). ^***^p < 0.001, control IgG versus CCL2 ab; ^††^*p* < 0.01, ^†††^*p* < 0.001, compared with the day 1 baseline threshold in control IgG group; ^###^*p* < 0.001, compared with the baseline threshold in CCL2 ab group; ^!!!^*p* < 0.001, compared with the day 20 threshold in the CCL2 ab group. **E** The effect of LD-IL-2 on sumatriptan-induced behavioral sensitization was abolished by the CCL2 neutralizing antibody in female C57BL/6J mice (*n* = 7/group). ^***^p < 0.001, control IgG versus CCL2 ab; ^†††^*p* < 0.001, ^###^*p* < 0.001, compared with the day 1 baseline threshold in control IgG and CCL2 ab groups, respectively; ^!!!^*p* < 0.001, compared with the day 20 threshold in CCL2 ab group

Results from our previous studies suggest that LD-IL-2 treatment increases the number/function of immunosuppressive Treg cells in dura and TG, thereby reversing chronic headache-related sensitization, including repeated sumatriptan-induced facial skin hypersensitivity [12, 20]. We have also found that all Treg cells in dura and TG express CCR2 [10]. The fact that CCL2 and CCR2 KO mice still exhibit sumatriptan-induced sensitization provides an opportunity to investigate whether CCL2-CCR2 signaling is functionally important for the therapeutic effect of LD-IL-2 (Fig. 5A). Male CCL2 KO and female CCR2 KO mice as well as wild-type controls were treated daily with LD-IL-2 (1 µg/mouse/day, i.p.) after the establishment of sumatriptan-induced facial skin hypersensitivity. In both male and female wild-type mice, LD-IL-2 normalized the facial mechanical thresholds in the presence of continuous sumatriptan administration (Fig. 5B-C) as we observed previously [12]. In contrast, LD-IL-2 was completely ineffective in CCL2 and CCR2 KO mice (Fig. 5B-C), indicating that the endogenous CCL2-CCR2 signaling pathway is required for LD-IL-2 to reverse MOH-related behavioral sensitization. When we used the neutralizing CCL2 ab to inhibit peripheral CCL2-CCR2 signaling during LD-IL-2 treatment, LD-IL-2 could no longer reverse sumatriptan-induced behavioral sensitization in either male or female mice (Fig. 5D-E), indicating that LD-IL-2 relies on peripheral CCL2-CCR2 signaling to reverse MOH-related behavioral sensitization.

**Fig. 6:**
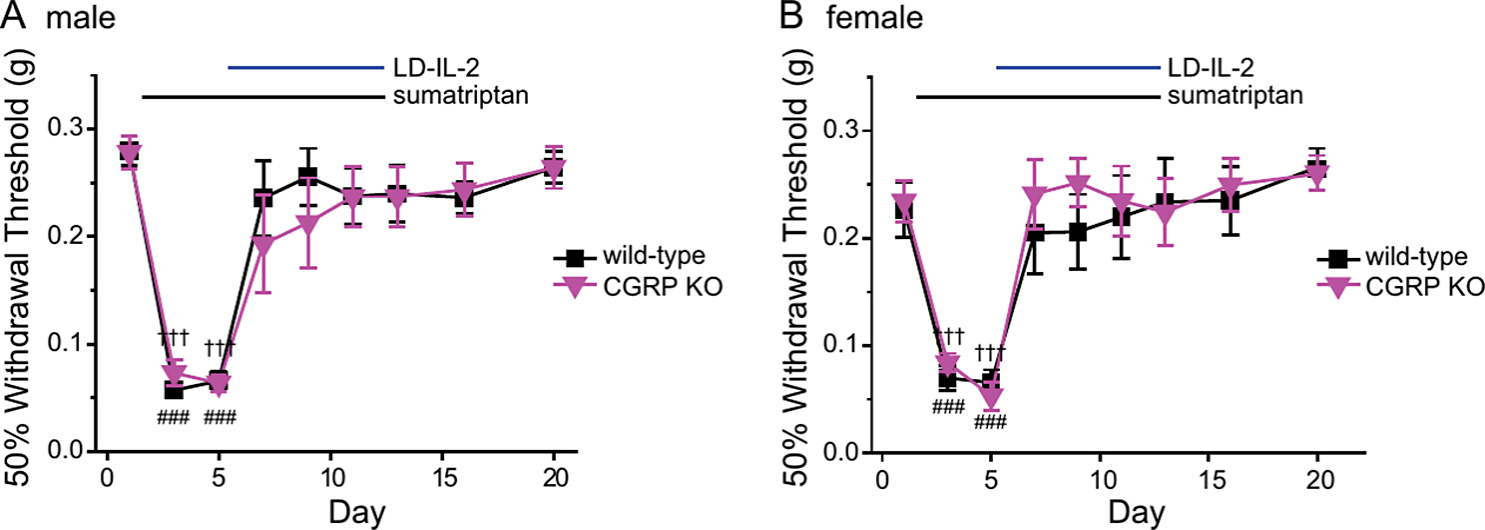
LD-IL-2 does not rely on CGRPα to reverse MOH-related behavioral sensitization. **A-B** LD-IL-2 reversed sumatriptan-induced facial skin hypersensitivity in male (**A**, *n* = 6–9/group**)** and female (**B**, *n* = 6–8/group**)** CGRPα KO mice. ^###^*p* < 0.001, ^†††^*p* < 0.001, compared with the baseline threshold within the wild-type and CGRPα KO groups, respectively

We also tested whether genetic loss of CGRPα affect the efficacy of LD-IL-2 treatment. Sumatriptan-induced acute sensitization was completely reversed by LD-IL-2 in male and female CGRPα KO mice, similar to what we observed in wild-type mice (Fig. 6A-B). Collectively, these data suggest that LD-IL-2 relies on CCL2-CCR2 signaling, but not the CGRP pathway to reverse MOH-related behavioral sensitization.

### CCL2-CCR2 signaling contributes to LD-IL-2-induced increase in Treg cells in dura and TG

**Fig. 7:**
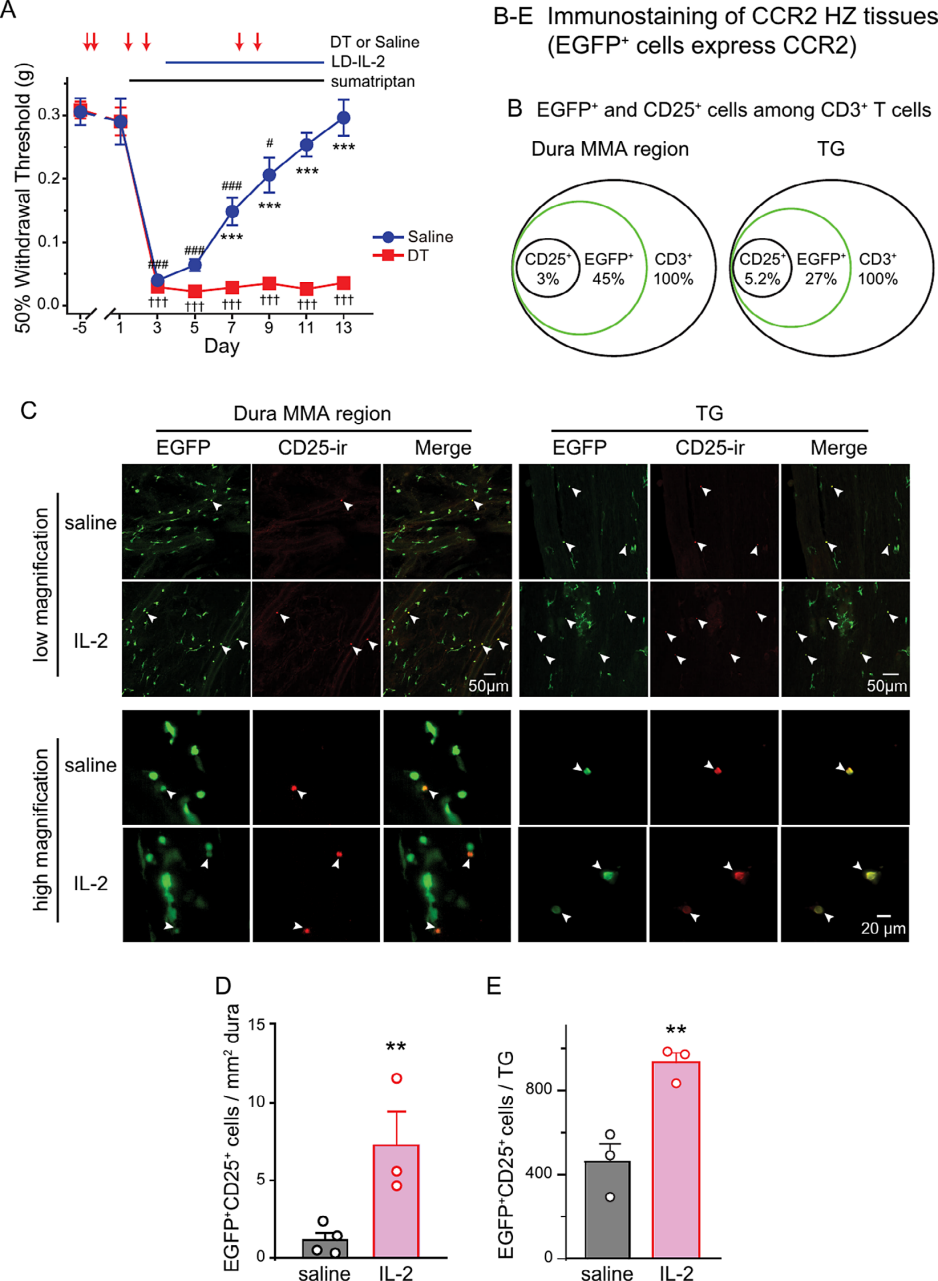
LD-IL-2 increases the number of CCR2-expressing, CD25^**+**^T cells in dura and TG. **A** Repeated diphtheria toxin (DT) administration abolished the effect of LD-IL-2 on sumatriptan-induced facial mechanical hypersensitivity in DEREG mice (saline group: 4 males and 4 females; DT group: 5 males and 3 females). ^***^p < 0.001, saline versus DT groups. ^#^*p* < 0.05, ^###^*p* < 0.001, compared with the day 1 threshold in saline group; ^†††^*p* < 0.001, compared with the day 1 threshold in the DT group. Note that all drugs were injected after the completion of behavioral tests on the same day. Both sumatriptan (day 1–2) and LD-IL-2 (day 3–12) were injected daily. **B** Venn diagrams of the percentages of EGFP^+^ and CD25^+^ cells among CD3^+^ T cells in dura surrounding the MMA and in TG tissues from naïve CCR2 HZ mice (replot of data published in [10]). **C** Representative images of EGFP^+^ and CD25^+^ cells in dura and TG tissues from CCR2 HZ mice after 12 daily injections of saline or LD-IL-2 (1 µg/mouse/day, i.p.). Arrowheads indicate CD25^+^ cells. **D-E** The density of EGFP^+^CD25^+^ cells in dura (**D**) and the number of EGFP^+^CD25^+^ cells in TG (**E**) of female CCR2 HZ mice after 12 daily injections of saline or LD-IL-2 (*n* = 3–4/group). ^**^p < 0.01, two-tailed t test

We used DEREG mice to test whether LD-IL-2 reverses MOH-related sensitization through endogenous Treg cells. The DEREG HZ mice contain a transgenic allele that selectively expresses DTR-EGFP fusion protein in Treg cells [16]. We treated DEREG mice with diphtheria toxin every 6 days to achieve long-term Treg cell depletion in dura and TG tissues [20]. Diphtheria toxin treatment did not alter the baseline mechanical thresholds (Fig. 7A, day − 5 versus day 1) or the magnitude of sumatriptan-induced sensitization (Fig. 7A, day 3). In saline-treated male and female DEREG mice with intact Treg cells, LD-IL-2 accelerated the resolution of sumatriptan-induced facial skin hypersensitivity, normalizing facial mechanical thresholds by day 11 (Fig. 7A). In contrast, the effect of LD-IL-2 was abolished in Treg-depleted mice of both sexes, and sumatriptan-induced sensitization persisted (Fig. 7A, day 3–13). These results confirm that the endogenous Treg cells mediate the therapeutic effects of LD-IL-2 on MOH-related behaviors.

We went on to examine CCR2 expression in Treg cells at baseline and after LD-IL-2 treatment in dura and TG tissues from CCR2 HZ mice, which express CCR2 from the wild-type allele and EGFP from the KO allele [15]. This allows us to use EGFP fluorescence to identify CCR2-expressing cells. Since the α subunit of IL-2 receptor CD25 is highly abundant in Treg cells, we used CD25-immunoreactivity to identify tissue Treg cells. We have reported in a previous study [10] that, at baseline, EGFP signal is present in 45% and 27% of CD3^+^ T cells in dura and TG of CCR2 HZ mice, respectively (Fig. 7B). About 3–5% of CD3^+^ T cells express CD25, thus are likely Treg cells (Fig. 7B). Notably, all CD25^+^ cells in dura and TG are EGFP^+^ under basal conditions (Fig. 7B), whereas in many tissues CCR2 is expressed in only a subset of Treg cells [23]. In the present study, we treated female CCR2 HZ mice with daily saline or LD-IL-2 (1 µg/mouse/day, i.p.) for 12 days and stained the whole-mount dura and TG sections with the anti-mouse CD25 antibody (Fig. 7C). LD-IL-2 treatment significantly increased the number of CD25^+^ cells in dura and TG tissues (Fig. 7C-E). Notably, most, if not all, CD25^+^ cells in dura and TG from both saline- and IL-2-treated mice were EGFP^+^ (Fig. 7C). These results indicate that LD-IL-2 treatment increases the number of CCR2-expressing Treg cells in mouse dura and TG, suggesting that the CCR2 signaling in Treg cells is functionally important to the therapeutic effects of LD-IL-2.

**Fig. 8:**
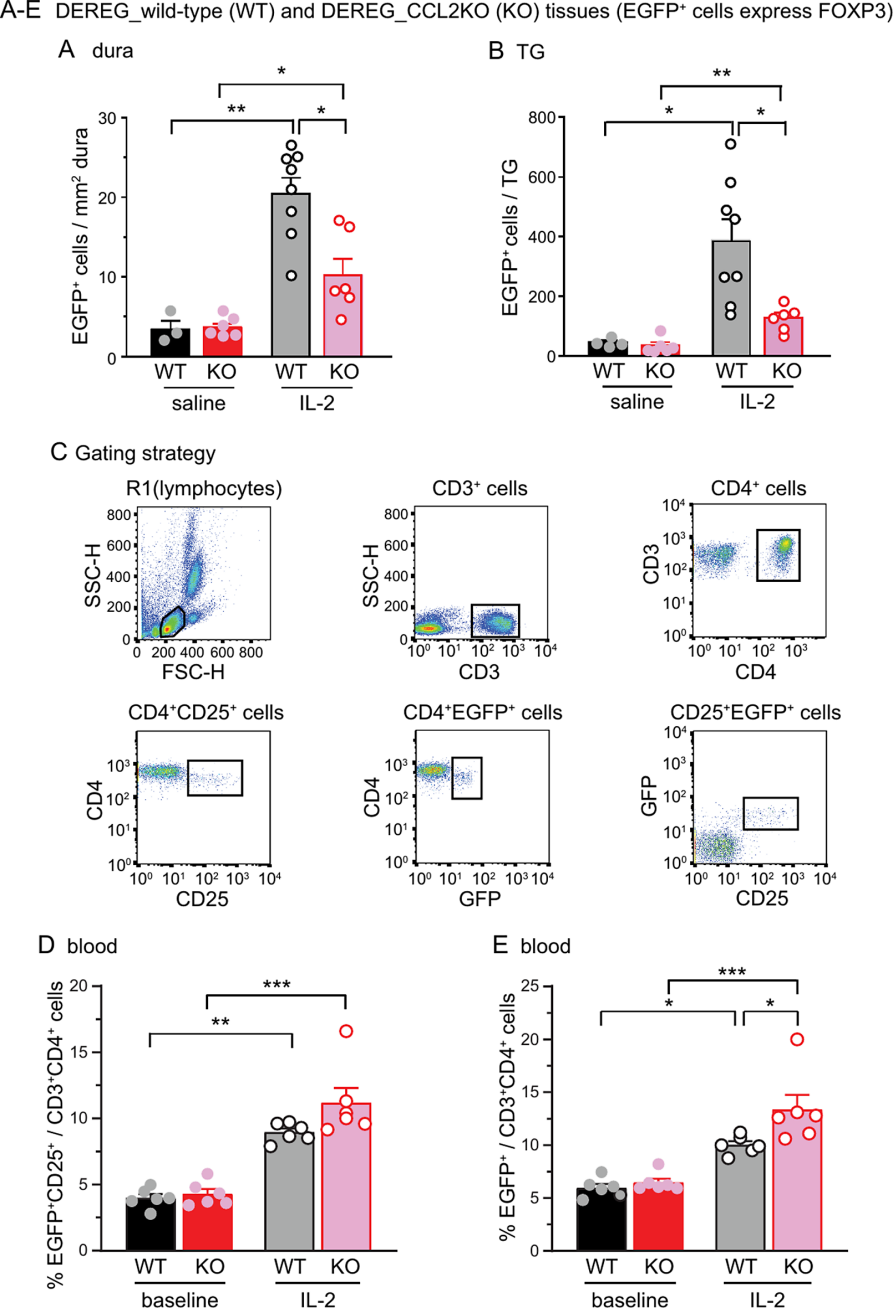
LD-IL-2-induced Treg cell increase in dura and TG is significantly reduced in CCL2 KO mice. **A** The density of EGFP^+^ Treg cells in dura surrounding the MMA from female DEREG (WT) and DEREG_CCL2KO (KO) mice at baseline and after 12 daily injections of saline or LD-IL-2 (*n* = 3–8/group). ^*^p < 0.05, ^**^p < 0.01. **B** The number of EGFP^+^ Treg cells in TG from DEREG and DEREG_CCL2KO mice at baseline and after 12 daily saline or LD-IL-2 (same mice as in **B**). ^*^p < 0.05, ^**^p < 0.01. **C** Gating strategy for individual T cell subpopulations. **D-E** The frequencies of EGFP^+^CD25^+^ (**D**) and EGFP^+^ (**E**) Treg cells in the peripheral blood from female DEREG and DEREG_CCL2KO mice at baseline and after 5 daily saline or LD-IL-2 injections (*n* = 6/group), respectively. ^*^p < 0.05, ^**^p < 0.01, ^***^p < 0.001

Next, we asked whether deletion of endogenous CCL2 affects LD-IL-2-induced Treg cell expansion. Since DEREG mice selectively express DTR-EGFP fusion protein in Treg cells [16], we crossed DEREG and CCL2 KO mice to generate the DEREG_CCL2KO line. This allowed us to use EGFP signal to identify Treg cells on wild-type and CCL2 KO background. In naïve mice, the number of Treg cells were comparable in dura and TG from female DEREG and DEREG_CCL2KO mice (Fig. 8A-B, WT + saline and KO + saline groups), indicating that CCL2-CCR2 signaling is not involved in controlling the number of tissue Treg cells under basal condition. After 12 daily LD-IL-2 treatments, there was a 6–8 fold increase in Treg cell numbers in the dural and TG from DEREG females (Fig. 8A-B, WT + IL-2 versus WT + saline), in line with the previous study [12]. In DEREG_CCL2KO females, the same LD-IL-2 regimen only induced a 2–3 fold Treg cell increases in dura and TG (Fig. 8A-B, KO + IL-2 versus KO + saline), significantly less than that in DEREG mice (Fig. 8A-B, WT + IL-2 versus KO + IL-2). These data point to a major role of the endogenous CCL2-CCR2 signaling in supporting LD-IL-2-induced Treg cell increase in dura and TG.

We went on to investigate whether CCL2-CCR2 signaling mediates LD-IL-2-induced expansion of Treg cells and/or the infiltration of Treg cells. We used flow cytometry analysis to address this question (Fig. 8C). The vast majority of EGFP^+^ Treg cells from naïve DEREG and DEREG_CCL2KO mice express IL-2 receptor CD25 (Fig. 8C), thus can respond to LD-IL-2 treatment. At baseline level, female DEREG and DEREG_CCL2KO mice had similar frequencies of EGFP^+^ and EGFP^+^CD25^+^ Treg cells among CD3^+^CD4^+^ T cells in peripheral blood (Fig. 8D-E, WT + baseline versus KO + baseline), indicating that CCL2-CCR2 signaling is not involved in controlling the number of circulating Treg cells under basal condition. LD-IL-2 treatment resulted in about a 2-fold increase in the frequency of EGFP^+^CD25^+^ cells in the blood from DEREG as well as DEREG_CCL2KO mice (Fig. 8D, WT + IL-2 versus KO + IL-2). Consequently, the frequency of total EGFP^+^ cells was significantly increased in both DEREG and DEREG_CCL2KO mice (Fig. 8E, WT + IL-2 versus KO + IL-2). These results suggest that CCL2-CCR2 signaling is not required for LD-IL-2-induced Treg cell expansion but plays a crucial role in mediating Treg cell infiltration to the dura and TG after LD-IL-2.

## Discussion

Although MOH and chronic migraine co-exist in many patients [1, 2, 11], whether they have common or distinct disease mechanism is not known. Our recent work shows that the peripheral CCL2-CCR2 signaling plays a pivotal role in the development of nitric oxide- and stress-induced persistent central sensitization that underlies headache chronification [10]. In the present study, both male and female mice developed robust behavioral sensitization in response to repeated sumatriptan administration, regardless whether they possess an intact or compromised CCL2-CCR2 signaling pathway. Thus, CCL2-CCR2 signaling is necessary for nitric oxide- and stress-induced sensitization but is not required for repeated sumatriptan-induced sensitization, indicating distinct molecular mechanisms underlying the pathophysiology of MOH and chronic migraine. We have found that CCR2 is expressed in macrophages and T cells but not in TG neurons [10]. It is possible that triptans can induce peripheral sensitization through activation of 5-HT_1B/1D_ receptors on primary afferent neurons without engaging CCL2-CCR2 signaling in immune cells.

We also found that, although CGRPα KO mice did not exhibit chronic migraine-related behaviors in response to repeated NTG administration [22], they developed sumatriptan-induced sensitization comparable to that of wild-type mice. At cellular level, repeated NTG administration significantly increases the number of CGRP-R and PACAP-R TG neurons [22], whereas repeated sumatriptan administration did not lead to similar enhancement of the CGRP and PACAP signaling pathways in TG neurons. These results support the notion that different molecular mechanisms mediate the development of MOH and chronic migraine, thus are of high clinical relevance. Our findings predict that inhibition of the CCL2-CCR2 or CGRP signaling pathway may alleviate chronic migraine but not MOH. In chronic migraine patients with medication overuse, antibodies that antagonize the CGRP signaling pathway likely work primarily through mitigating the primary chronic migraine, thereby reducing the medication overuse [24–27].

Our finding is consistent with the report that the CGRP receptor antagonist olcegepant does not reverse MOH-related sensitization in mice after chronic exposure to morphine and nitric oxide donor [9]. However, it is in contrast with previous studies indicating the functional importance of CGRP signaling in a rat model of MOH [5, 7, 8]. More work is needed to determine whether the differences in the sumatriptan administration regimen (daily injection versus chronic infusion) and/or the species (mice versus rats) account for the contrasting outcomes. Future in-depth studies are also warranted to investigate whether CCL2-CCR2 and CGRP signaling pathways contributes to MOH associated with other acute headache medications, for example, barbiturates and opioids. Notably, in both chronic migraine with MOH patients and in the rat model, overuse of non-steroidal anti-inflammatory drugs (NSAIDs) disrupts intestinal barrier function and increases the levels of pro-inflammatory mediators in the circulation, thereby causing low-grade inflammation [28, 29]. Whether CCL2-CCR2 and CGRP signaling are involved in NSAIDs-induced MOH merits further study.

In both male and female mice, LD-IL-2 treatment not only reverses NTG and sumatriptan-induced persistent facial skin hypersensitivity, but also prevents the development of hyperalgesic priming, which is mechanistically related to the transition from episodic to chronic headache [12]. Importantly, repeated sumatriptan-induced hyperalgesic priming may also be mechanistically related to the development of withdrawal headaches in the acute phase of drug discontinuation during MOH treatment. Together, these findings in mouse models suggest that LD-IL-2 may be particularly useful in patients with chronic migraine and medication overuse, especially for those that experience withdrawal headaches.

The mechanism of action of LD-IL-2 is likely through increasing the number/function of Treg cells in dura and TG, thereby reversing the sensitization of TG neurons through both interleukin-10 and transforming growth factor beta 1 signaling pathways [12, 22, 30]. Surprisingly, LD-IL-2 treatment was ineffective for sumatriptan-induced facial skin hypersensitivity in mice with defective CCL2-CCR2 signaling, suggesting that LD-IL-2 engages endogenous CCL2-CCR2 signaling pathway to reverse MOH-related behavioral sensitization.

CCL2 is expressed in TG neurons as well as mural cells closely associated with dural blood vessels during normal and chronic migraine-related states [10]. In mice with MOH-related persistent sensitization, CCL2 is likely released from activated TG neurons and perivascular mural cells. This, in turn, would create a CCL2 concentration gradient that attracts CCR2-expressing immune cells, including a subpopulation of circulating Treg cells that express CCR2 [31].

Although CCR2 signaling contributes to leukocyte egress from bone marrow, the absolute number of T cells in peripheral blood is not altered by genetic deletion or pharmacological inhibition of CCR2 in mice and humans [32, 33]. The frequency of Treg cells is also comparable in tissues from wild-type and CCR2 KO mice [34]. In line with these findings, we found that the abundance of Treg cells is comparable in peripheral blood, dura and TG tissues from naïve wild-type and CCL2 KO mice. After LD-IL-2 treatment, the frequency of Treg cells in blood was doubled in both DEREG and DEREG_CCL2KO mice. This is not surprising, given that CCR2 is only expressed in 10–20% of Treg cells in the peripheral blood and 7–8% of Treg cells in lymph nodes [31]. In contrast, Treg cell increase in dura and TG was greatly subdued in DEREG_CCL2 KO mice after LD-IL-2 treatment. These results indicate that the CCL2-CCR2 signaling pathway is not required for LD-IL-2-induced Treg expansion. Rather it is critically important for the infiltration of the expanded Treg cells to dura and TG tissues, consistent with the previous work showing that the migration of Treg cells from blood to the inflamed allograft is CCR2-dependent [35].

In the absence of CCL2, LD-IL-2 treatment still led to 2–3 fold increase in Treg cell numbers in dura and TG. This may result from the proliferation of resident Treg cells. It is also possible that, in addition to CCR2, other chemokine receptors such as CCR4 and CCR5 mediate Treg cell migration from blood to dura and TG [35]. Notably, despite the moderate Treg cell increase, the therapeutic benefit of LD-IL-2 was completely lost in CCL2 KO mice. This is reminiscent of prior studies indicating that CCR2 signaling increases the fitness of Treg cells, and CCR2 deficiency renders Treg cells functionally inferior to wild-type counterparts in suppressing autoimmunity after they are locally transferred to allograft tissue sites [34, 35]. Future studies are needed to determine whether the CCL2-CCR2 signaling pathway plays a non-chemotactic role for Treg cells to reverse headache-related sensitization.

We have shown that LD-IL-2 treatment reverses chronic headache-related behaviors in mouse models of chronic migraine, post-traumatic headache and MOH [12]. It is likely that the peripheral CCL2-CCR2 signaling mediates the therapeutic effects of LD-IL-2 on all occasions. In human memory Treg cells, CCR2 is expressed along with other migration and homing receptors [36], and may contribute to the tissue infiltration of Treg cells in future clinical studies testing the efficacy of LD-IL-2 on human chronic headache disorders.

One limitation of this study is that we modeled sumatriptan-induced sensitization and its resolution by LD-IL-2 in mice that have not been repeatedly exposed to triggers of primary headache, whereas MOH patients usually have a long history of migraine or tension-type headache [1, 2]. Although this allows us to reveal how CCL2-CCR2 and CGRP pathways differentially contribute to the establishment and the resolution of chronic migraine and MOH, our conclusions need to be verified in additional preclinical models that better capture the complexity of chronic headache patients with medication overuse. Another limitation is that estrous cycle analysis was not performed, which may confound data obtained from female mice. Lastly, we tested the effects of LD-IL-2 on MOH-related behaviors in female but not male CCR2 KO mice (Fig. 5C). In our previous study [10] as well as in this work (Figs. 2 and 5), mice with CCL2 or CCR2 dysfunction displayed the same deficits (or the lack of). For example, neutralizing CCL2 (Fig. 5D-E) or genetic deletion of CCR2 (Fig. 5C) ll abolished the therapeutic benefits of LD-IL-2. Moreover, we did not observe any sex-dependent differences between mice with CCL2 or CCR2 dysfunction. For example, neutralizing CCL2 in both male and female wild-type mice negated the effects of LD-IL-2 (Fig. 5D-E). We therefore reason that it is likely that LD-IL-2 will lose its therapeutic effects on MOH-related sensitization in male CCR2 KO mice, same as what we observed in female CCR2 KO, male CCL2 KO as well as male and female mice receiving the CCL2 neutralizing antibody. That said, we acknowledge the limitation of not directly testing the effects of LD-IL-2 on MOH-related behaviors in male CCR2 KO mice due to the limited number of CCR2 KO mice produced in our colony during the course of this study.

## Conclusions

In summary, results from our prior work and the present study provide evidence that distinct molecular mechanisms underlie chronic migraine and MOH. We have revealed the dual role of the CCL2-CCR2 signaling pathway: on one hand facilitating headache chronification through enhancing both CGRP and PACAP signaling pathways in TG neurons [10], while on the other hand accelerating the resolution of chronic headache by LD-IL-2 through enhancing the infiltration/functionality of Treg cells. This expands our knowledge on the mechanism of action of LD-IL-2 treatment in reversing chronic headache-related sensitization.

## Data Availability

No datasets were generated or analysed during the current study.

## Abbreviations

5-HT_1B/1D_ receptors: Serotonin 1B and 1D receptors
CCL2: C-C motif ligand 2
CCL2 ab: Anti-mouse CCL2 antibody
CCR2: C-C motif chemokine receptor 2
CGRPα: Calcitonin gene-related peptide-α
CGRP-R: CGRP-responsive
DEREG: Depletion of regulatory T cell
DTR-EGFP: Diphtheria toxin receptor-EGFP
EGFP: Enhanced green fluorescent protein
HZ: Heterozygous
i.p: Intraperitoneal
KO: Knockout
LD-IL-2: Low-dose interleukin-2
Min: Minute
MMA: Middle meningeal artery
MOH: Medication overuse headache
NGS: Normal goat serum
NSAIDs: Non-steroidal anti-inflammatory drugs
NTG: Nitroglycerin
PACAP: Pituitary adenylate cyclase–activating polypeptide
PACAP-R: PACAP-responsive
PB: Phosphate buffer
PBS: Phosphate buffered saline
PCR: Polymerase chain reaction
Qpcr: Quantitative PCR
RM ANOVA: Repeated measures analysis of variance
ROI: Region of interest
RT: Room temperature
TG: Trigeminal ganglion
Treg cell: Regulatory T cell
